# Multimorbidity Progression among Medicare Beneficiaries in the Health and Retirement Study (1992-2014)

**DOI:** 10.1093/geroni/igab046.2347

**Published:** 2021-12-17

**Authors:** Ana Quiñones, Sheila Markwardt, Heather Allore, Jason Newsom, Corey Nagel, David Dorr, Anda Botoseneanu

**Affiliations:** 1 Oregon Health & Science University, Portland, Oregon, United States; 2 Yale School of Medicine, New Haven, Connecticut, United States; 3 Portland State University, Portland, Oregon, United States; 4 University of Arkansas for Medical Sciences, Little Rock, Arkansas, United States; 5 University of Michigan - Dearborn, Dearborn, Michigan, United States

## Abstract

Older adults are at greater risk for developing and accumulating multimorbidity, defined as 2 or more chronic diseases. This study describes the characteristics of multimorbidity progression-based groups using Medicare claims chronic condition warehouse algorithms over a 24-year period. The HRS-Medicare linked data (1991-2015, N=17,895, age 67 years and older) were used in descriptive analyses presented as a Sankey flow diagram. We identified 1,293 (7.2%) beneficiaries who had not yet developed multimorbidity by the end of the observation period (no multimorbidity), 7,552 (42.2%) who started without but developed multimorbidity over the course of observation (incident multimorbidity), and 9,050 (50.6%) who had multimorbidity upon study entry (prevalent multimorbidity). There were notable differences between multimorbidity progression-based groups. Beneficiaries with prevalent multimorbidity were younger at baseline (73.1% in youngest age category [67-69] vs. 50.3% for incident and 66.7% for no multimorbidity), had proportionately higher levels of cognitive impairment (21.6% CIND/dementia vs. 15.4% for incident and 16.8% for no multimorbidity), and greater mean levels of functional impairment and healthcare utilization. Non-Hispanic Black beneficiaries were more represented in prevalent multimorbidity (15.4%) than in the incident (11.8%) and no multimorbidity groups (13.4%). Non-Hispanic White beneficiaries were more represented in the incident (83.5%) than the prevalent (77.2%) and the no multimorbidity (77.7%). Hispanic beneficiaries were more represented in the no (8.9%) than the prevalent (7.4%) and incident multimorbidity groups (4.7%). Results highlight beneficiaries who experience clinically-meaningful transitions to multimorbidity states in late life, allowing new insights and informing interventions to address burdensome changes to their chronic disease status.

